# Palliative care, unpaid care and deprivation in Scotland: a study using census and vital registration data.

**DOI:** 10.23889/ijpds.v7i3.2034

**Published:** 2022-08-25

**Authors:** Iain Atherton, Michelle Jamieson, Jan Savinc

**Affiliations:** 1 Edinburgh Napier University

## Approach

The analysis was based on a linkage of the Scottish 2011 census and death registration records where deaths had occurred within 12 months of the decennial census. Areal deprivation used the Scottish Index of Multiple Deprivation, a widely used indicator that uses administrative data to rank areas by degree of deprivation. Palliative care needs were assessed using a validated approach that utilizes ICD10 codes from death registrations. Availability of unpaid care was assessed using place of residence at point of census and indication of other people resident in the same household.

## Results

There were 52,553 death records of which 46,473 were for people aged 18 years or older at census and had matched census records. Deaths with palliative care needs were highest in the least deprived areas, though proportions were high regardless of deprivation ranging from 81.2% of deaths in the least deprived to quintile to 76.0% in the most deprived. Given the higher number of deaths in the most deprived areas, numbers of deaths were spread evenly across quintiles. In contrast, people approaching the end of life at point of census were more likely to live alone if resident in the most deprived quintile at point of census (40.8% vs 26.5%).

## Conclusion

Palliative care needs are high regardless of levels of deprivation. However, it may be that those residing in more deprived areas not only have fewer financial resources to cope but also have lower recourse to support within the household.

